# Associations between domains of sedentary behavior, well-being, and quality of life – a cross-sectional study

**DOI:** 10.1186/s12889-024-19252-9

**Published:** 2024-07-02

**Authors:** Sabrina C. Teno, Marlene N. Silva, Pedro B. Júdice

**Affiliations:** 1 Faculdade de Educação Física e Desporto, CIDEFES, Universidade Lusófona, Campo Grande, Lisbon, Portugal; 2 Programa Nacional Para a Promoção da Atividade Física, Direção-Geral da Saúde, Lisbon, Portugal; 3 CIFI2D Universidade Do Porto, Porto, Portugal

**Keywords:** Sedentary behavior, Domain specific, Well-being, Psychological well-being, Quality of life

## Abstract

The importance of reducing sedentary behavior (SB) in the prevention of mortality and chronic and mental diseases is scientifically well grounded, but SB can be accumulated in diverse domains of life, such as leisure-time SB, transport between home/work/school when sitting (transport-related SB), or in occupational settings such as working or studying (occupational SB), and the associations for each domain of SB with well-being measures and quality of life are still underexplored from a positive perspective. Through a cross-sectional investigation, we collected data from 584 participants who completed a questionnaire throughout November 2021 and with Spearman correlation test, analysed the associations between SB in three different domains with psychological well-being, satisfaction with life, and quality of life. Our results indicated that after adjustment for physical activity, sex, body mass index, smoking history, chronic disease status, financial perception, quality/duration of sleep and university group, in younger adults (18 to 24 years old), leisure-time SB was negatively related to psychological well-being (rho = -0.255; *p* = 0.008), and in adults (25 to 64 years old), occupational SB was negatively related to satisfaction with life (rho = -0.257; *p* < .001) and the mental component of quality of life (rho = -0.163; *p* = 0.027). Our findings highlight the idea that not all SB is built the same and that future strategies to reduce SB from people’s lives must target specific domains of SB according to the age group when aiming to improve well-being and quality of life.

## Background

The importance of reducing sedentary behavior (SB) in the prevention of chronic diseases and mortality [1] is scientifically well grounded, and recently, interest in the relation between SB and mental disorders has been growing [2–4], since both have increased in the past decade[5, 6]. SB is characterized by a series of activities in a sitting or reclining posture with low energy expenditure (≤ 1.5 metabolic equivalents [MET]) while awake [7], such as the use of electronic devices (e.g., television, computer, tablet, phone); reading/writing/talking while sitting; and sitting in a bus, car or train. In cross-sectional investigations, excessive SB accumulation has been associated with mental health problems (i.e., anxiety, depression, and suicidal thoughts in colleges [8], anxiety and depression in adults [9], and symptoms of depression in older adults [10]). A direct association between SB and the risk of depression has also been observed [2]. However, sedentary pursuits can be accumulated in diverse domains of life, such as leisure-time SB, including watching TV or using social media [11], transport between home/work/school when sitting (transport-related SB), or in occupational settings such as working or studying (occupational SB), but investigations about their prevalence and its relation with mental health in adults are scarce [12–14].

Recently, investigations have been looking into this topic from a positive perspective, that is, not analysing the associations with mental disorders but also evaluating the relationship with mental well-being [15]. There is no consensus around a single definition of well-being, but there is a general agreement that includes the presence of positive emotions and moods (e.g., contentment, happiness), the absence of negative emotions (e.g., depression, anxiety), satisfaction with life and positive functioning [16, 17]. This definition includes a hedonic and evaluative aspect (affects and life satisfaction, used here as subjective well-being) and a eudaimonic feature (self-acceptance, autonomy, control under the environment, positive relationships, purpose in life and personal development) – used as psychological well-being [18, 19]. Quality of life is another form to assess people’s well-being; therefore, the World Health Organization (WHO) defines quality of life as an individual’s perception of their position in life in the context of the culture and value systems in which they live and about their goals, expectations, standards, and concerns [20]. On the individual level, quality of life has been assessed through health-related quality of life (HRQoL) and includes physical and mental perceptions [21].

The prevalence of SB accumulation among different domains has rarely been explored from this perspective, and it can be of relevance. For example, younger adults (i.e., 18 to 24 years) and older adults (i.e., ≥ 65 years) seem to be slightly more sedentary in their leisure time [13, 14], while adults spend more time in occupational SB. An investigation with university students (mean age 20 [± 1.5]) found that they spent over 12 hours/day in SB, with a third of this time being spent in leisure time [22]. Another investigation [23] conducted in university students (mean age 21 [± 1.8]) found that weekday SB was positively related to one dimension of quality of life (intimacy), while SB on weekends was negatively related to some dimensions of quality of life (intimacy, safety and communicative with others). On the other hand, a study focusing on university employees [24] (mean age 42 [± 9] indicated that longer sitting time on weekdays was associated with decreased mental well-being, suggesting that, in addition to the context, age group can have some influence on the relationship between SB and well-being or quality of life.

Thus, although the current recommendations about SB are “"sit les”" or “minimize time sitting in front of screens” from the WHO [25], we believe that in regards to well-being and quality of life, it is prudent to further understand how each domain of SB can relate to people’s well-being so that the recommendations can be more accurate [26]. Therefore, this study aims to evaluate the cross-sectional associations for three SB-specific domains (i.e., leisure, occupational, and transport-related) with measures of well-being and quality of life and whether it differs between a sample of younger and older adults from a Portuguese university.

## Methods

### Design

This is a cross-sectional investigation with data from the LusófonAtiva Project that aimed to design and implement a digital system for monitoring physical behaviors linked to healthy lifestyles (ILIND/F + /EI/02/2020). This study was developed and implemented at the largest private university in Portugal. Participants were recruited online via convenience sampling through invitations via email and university social networks with a link to the questionnaire (via the Qualtrics platform) throughout November 2021.

### Study population

This is a subsample from the 791 participants from the university campus who volunteered to participate, as for these analyses, we only included data from 584 participants who completed the questionnaire considering the main variables of the current study.

### Measures

#### Sedentary behavior

Total SB was assessed with the short version of the International Physical Activity Questionnaire (IPAQ) [27]. The IPAQ was chosen because it is an internationally validated questionnaire that has already been tested in several countries and demonstrated acceptable properties of reliability and validity, especially in urban samples [27]. However, to capture the domains of SB, questions were created regarding the time that participants usually sat at work/school (occupational SB), on transport (transport-related SB), and in their leisure time. The questions were as follows: “On average, how much time do you usually spend sitting at study/work per day?”, “On average, how much time do you usually spend sitting making a hobby, watching TV, using the computer/tablet/cell phone for leisure per day”, and “On average, how much time do you spend sitting on public/private transport per day?”. The answers were weighted by day [min/day]) through the weighted arithmetic media of the responses of the time on weekdays and weekend days in each domain of SB and were used as continuous variables.

#### Well-being

Following recent guidelines for the selection of well-being measures [28], a set of items representative of each aspect of subjective well-being was created. They presented themselves on scales from 0 "not satisfied" to 10 "completely satisfied" about work/course, personal relationships, and free time to do what they like (assessing the facets of hedonics and evaluative of well-being). It was calculated as a mean of these 3 items and used here as “general life satisfaction” [GLS]). In addition, to assess the eudaimonic facet of well-being, the following question was used: On a scale from 0 to 10, how much do you agree with these statements: “I feel that my life and the things I do are worthwhile” and “I feel my life has purpose and direction”, used here as “psychological well-being” (PWB), and then the mean of 2 items was calculated, with higher values indicating more wellbeing in both cases.

#### Quality of life

It was evaluated through the 12-item Short Form Survey (SF-12) [29]. It is a self-reported method that assesses the health impact on an individual's everyday life, and it is an abbreviated version of its predecessor, the SF-36 [30], with 36 items. The SF-12 contains a subset of 12 items of the SF-36, with one or two items from each of the eight original questionnaire scales, which is considered too long for large group comparisons and longitudinal monitoring. The information of all 12 items is used to construct a summary measurement of physical and mental components without losing their reliability. For this analysis, only the measure of mental component was used: 1 item of energy/fatigue (During the last month, have you had a lot of energy?); 1 item of social functioning (To what extent, in the last month, has your physical health or emotional issues limited your social activity, such as being—even digitally—with close friends or family?); 2 items of role-emotional (As a consequence of your emotional state, a) did you do less work/study than you would like? b) Performed work or other activities less carefully than usual?); and 2 items of mental health (During the last month, did you feel blue/calm and quit?). These questions are identified in the mental component of the Short Form Survey (MCS-12), which is a valid measure of mental health in epidemiological research and a useful screening tool for both depression and anxiety disorders [31]. The response options were “never” (5 points), “shortly” (4 points), “some time” (3 points), “the most part of the time” (2 points), and “always” (1 point). The questions were coded so that the highest score indicated better mental health and were used as continuous variables (ranging from 6 to 30 points).

### Covariates

Based on previous evidence about the associations of body composition [32], sex [33], age [34], chronic diseases [35], tobacco smoking [36], financial situation [37], physical activity [38], and sleep disorders [39] with depression (i.e., worse well-being), the following covariates were included in the statistical models.

#### Body mass index (BMI)

BMI was calculated as weight/height^2^ [kg/m^2^], according to the self-reported body weight and height, and used as a continuous variable.

#### Age and sex

Self-reported and included as continuous and categorical variables, respectively (i.e., sex: male, female, or other).

#### Smoking history

It was assessed through the question “Have you ever smoked?”, with three response options: "Never smoked"; "I have smoked, but I do not currently smoke"; and "currently smoking”), which was used as a categorical variable.

#### Sleep duration

The amount of sleep was assessed by the question: "In a typical week, what time do you usually lie down at night and get up in the morning?". The results (in hours) were used as a continuous variable.

#### Sleep quality

Sleep quality was assessed by the mean of the answers to the following questions: 1. "In a typical week, do you have trouble sleeping? 2. "In a typical week, do you have trouble staying awake during meals, classes/work while driving or participating in a social activity?". The answer options were as follows: "never" (4 points); "less than once a week" (3 points); "1 or 2 times a week" (2 points); and "3 or more times a week" (1 point), with more points indicating a better quality of sleep, ranging from 2 to 8 points. The variable was used as a continuous variable.

#### Presence or absence of chronic disease

This was assessed through the question “Do you have any chronic illness (physical or psychological) or disability diagnosed by a doctor?” with two response options: “Yes or not”, which was used as a dichotomic variable.

#### Financial situation

This evaluation was made through the personal perception of the current financial situation. It was assessed through the question: “How do you classify your current financial situation?” ("Difficult" and "Very difficult" [classified as “low financial perception”; "sufficient to pay the bills" [classified as “medium financial perception”], "comfortable" and "very comfortable"[ classified as “high financial perception”]), which was used as a categorical variable.

#### Physical activity (PA)

PA was assessed with the short version of the IPAQ, and the participants were categorized as “meeting the recommended PA goal” (≥ 150 min/week of moderate PA or ≥ 75 vigorous) or “not meeting the recommended PA” (< 150 min/week of PA moderate or < 75 vigorous) according to the guidelines for PA from the WHO [25] and used as a categorical variable.

### Statistical analysis

The data analyses were performed using the software Jamovi project (2021), version 2.2, and the significance level was maintained at 5%. Data normality was verified by the Shapiro‒Wilk test. Sample characteristics are presented as the mean and standard deviation for the continuous variables and as frequencies for the categorical variables. Analyses were performed just with complete data, i.e., without imputed values.

We separated our sample into groups of young adults (18 to 24 years) and adults (≥ 25 to 64 years), and to test the differences between groups, we used the Mann‒Whitney test, except for GSL, which we used the independent T test, since it presented a normal distribution. The chi-square test was used to verify the difference in the distribution of the responses between groups regarding the categorical variables. The associations between each specific domain of SB and GLS, PWB, and MCS were examined using the Spearman correlation test.

## Results

### Overall results

The total sample (*n* = 584) consisted of 68 employees (11.6%), 111 teachers (19.0%), and 405 students (69.3%), and the characterization of the sample is described in Tables 1 and 2.

**Table 1:** Characterization of the sample’s continuous variables

Variables	N	% of total sample	Mean	Standard deviation	*P* value T test or Mann–Whitney
Age					
Young adults (18 to 24 years)	262	44.8	20.4	± 1.9	
Adults (25 to 64 years)	322	55.1	41.2	± 10.2	** < .001**
Body mass index (BMI) kg/m^2^					
Young adults	262	44.8	23.1	± 4.3	
Adults	322	55.1	24.8	± 4.7	** < .001**
Sleep duration (h)					
Young Adults	167	22.2	7.5	± 1.5	
Adults	236	31.0	7.3	± 1.3	**0.011**
Sleep quality (2 to 8 points)					
Young adults	167	22.2	2.7	± 0.8	
Adults	235	40.2	3.0	± 0.7	** < .001**
Sedentary behavior total (min/day)					
Young adults	262	44.8	393.8	± 135.2	
Adults	322	55.1	357.9	± 135.9	**0.001**
Sedentary behavior – Domains (min/day)					
Leisure-time SB					
Young adults	165	28.2	269.6	± 161.6	
Adults	238	40.7	170.9	± 112.0	** < .001**
Transport-related SB					
Young adults	169	28.9	86.3	± 58.9	
Adults	237	40.5	65.5	± 53.9	** < .001**
Occupational SB					
Young adults	168	28.7	466.0	± 200.3	
Adults	236	40.4	409.1	± 189.0	** < .001**
Well-being measures					
GLS (mean of 3 items – 0 to 10 points)					
Young adults	101	13.5	5.0	± 2.1	
Adults	206	27.0	6.3	± 1.9	< .001*
PWB (mean of 2 items – 0 to 10 points)					
Young adults	134	22.9	6.6	± 2.5	
Adults	210	35.9	7.9	± 1.9	** < .001**
MCS (6 to 30 points)					
Young adults	132	22.9	19.5	± 5.2	
Adults	209	35.7	23.2	± 4.2	** < .001**

Abbreviations: *GLS* general life satisfaction, *PWB* psychological well-being, *MCS* mental component of short form survey-12, *SB* sedentary behavior. Bold: *p* < 0.05; (*) T-test

**Table 2:** Characterization of the sample’s categorical variables

	**Young adults (N)**	**% of the total sample**	**Adults (N)**	**% of the total sample**	***P*** ** value** **Chi-square**
**Sex**					
Female	182	31.1	191	32.7	
Male	75	12.8	130	22.2	
Other	5	0.6	1	0.1	**0.003**
Smoking history					
Never smoked	91	15.5	121	20.7	
I have smoked, but currently I do not	25	4.2	56	9.5	
Currently smoking	19	3.2	35	5.9	0.138
Financial situation perceived					
Low	39	6.6	41	7.0	
Medium	135	23.1	174	29.7	
High	88	15.0	107	18.3	0.717
Do you have some chronic disease?					
Yes	25	4.2	41	7.0	
Not	107	18.3	168	28.7	0.877
Met the physical activity recommendations					
Yes	162	27.7	194	33.2	
No	86	14.7	104	17.8	0.957

Bold: *p* < 0.05

As expected, Tables 1 and 2 show that there were several differences between age groups both in the independent and dependent variables, justifying a deeper investigation (see item 3.2).

Table 3 shows the raw associations between SB domains and well-being measures (GSL, PWB and MCS-12) and the adjusted models.

**Table 3:** Correlations for sedentary behavior domains with well-being measures and quality of life for the entire sample

	**Unadjusted model**			
		Leisure-time SB	Occupational SB	Transport-related SB
GLS	Spearman’s rho	-0.091	-0.292***	-0.136*
	*p* value	0.117	** < .001**	**0.018**
	N	300	300	302
PWB	Spearman’s rho	-0.172**	-0.166**	-0.152**
	*p* value	**0.002**	**0.002**	**0.005**
	N	336	336	337
MCS-12	Spearman’s rho	-0.215***	-0.191***	-0.072
	*p* value	** < .001**	** < .001**	0.189
	N	333	333	334
	**Adjusted model**			
GLS	Spearman’s rho	0.046	0.232***	-0.052
	*p* value	0.453	** < .001**	0.397
	N	278	278	281
PWB	Spearman’s rho	-0.040	-0.087	-0.098
	*p* value	0.490	0.132	0.091
	N	310	310	312
MCS-12	Spearman’s rho	-0.040	-0.059	0.039
	*p* value	0.486	0.312	0.495
	N	310	310	312

^*^p < .05; **p < .01; ***p = < .001

Adjusted model for age, sex, compliance with PA guidelines, BMI, smoking history, chronic disease status, financial perception, sleep (quality and duration), and university group (student, teacher, or staff)

Abbreviations: *GLS* general life satisfaction, *PWB* psychological well-being, *MCS-12* mental component of short form survey-12, *SB* sedentary behavior

The analyses for the overall sample indicated that two of the three domains of SB were negatively and significantly associated with GLS (transport-related SB [*p* = 0.018] and occupational SB [*p* = < 0.001]). Furthermore, the three domains of SB were negatively associated with PWB (leisure-time SB and occupational [both, *p* = 0.002], and transport-related SB [*p* = 0.005]), and two were negatively associated with MCS-12 (occupational SB and leisure-time SB – both *p* < 0.001). In the adjusted model, the associations were attenuated, and only occupational SB remained negatively associated with GLS (*p* < 0.001), as described in Table 3.

### Subgroup analysis

As differences were found between younger adults and adults for both the independent and dependent variables (see Tables 1 and 2), we examined the correlations between SB domains and measures of well-being and quality of life while separating the two age groups (Table 4).

**Table 4:** Correlations for sedentary behavior domains with measures of well-being and quality of life separated by age group

		Leisure-time SB	Occupational SB	Transport-related SB
**Young adults**	**(18 to 24 years old)**			
GLS	Spearman’s rho	0.023	-0.198	0.026
	*p* value	0.843	0.078	0.819
	N	88	89	91
PWB	Spearman’s rho	-0.255**	-0.052	-0.011
	*p* value	**0.008**	0.595	0.907
	N	116	117	118
MCS-12	Spearman’s rho	-0.150	0.053	0.185
	*p* value	0.124	0.583	0.054
	N	116	117	118
**Adults**	**(25 to 64 years old)**			
GLS	Spearman’s rho	0.045	-0.257***	-0.097
	*p* value	0.551	** < .001**	0.195
	N	190	189	190
PWB	Spearman’s rho	0.013	-0.107	-0.176*
	*p* value	0.857	0.149	**0.017**
	N	194	193	194
MCS-12	Spearman’s rho	-0.027	-0.163*	-0.067
	*p* value	0.713	**0.027**	0.363
	N	194	193	194

*p < .05; **p < .01; ***p = < .001

Adjusted model for compliance with PA guidelines, sex, BMI, smoking history, chronic disease status, financial perception, quality, duration of sleep and university group (student, teacher, or staff)

Abbreviations: *GLS* general life satisfaction, *PWB* psychological well-being, *MCS-12* mental component of short form survey-12, *SB* sedentary behavior

## Discussion

To the authors’ knowledge, research on the relationship between distinct SB domains and mental health outcomes through a positive perspective is very scarce [40]. We found that SB was highly prevalent among the participants in the occupational pursuits. Our data indicated that all the domains of SB were negatively associated with PWB. Occupational SB and transport-related SB were negatively correlated with GLS, and leisure-time SB and occupational SB were negatively associated with MCS-12 in the unadjusted mode. In the adjusted model, the associations were attenuated, and only occupational SB remained negatively associated with GLS. Thus, taken together with the results of the present investigation, our findings differ from those of Scarabottolo et al. (2022) [40], where it was reported that screen time (i.e., watching television, using computers, and cell phones – without clarifying if for leisure or occupational purposes) may be negatively associated with social functioning (component of HRQoL) but positively related to other components (i.e., physical functioning, role-physical and role-emotional) after two years of follow-up.

Although the mechanisms linking SB with mental health are still unknown, several hypotheses may be suggested. First, SB takes time away from PA, which in turn is responsible for the prevention and treatment of diseases or conditions that impair well-being (such as depression and anxiety) [41], possibly due to the mediation of biological factors (such as interleukin 6 and brain-derived neurotrophic factor [BDNF] release during exercise). In addition, there are clinical factors (such as global functioning and frequency of physical symptoms), psychological factors (life satisfaction and self-esteem) and social factors (such as social support and marital status) associated with PA [42] that can also affect mental health. Second, longer sitting time has been associated with social isolation (i.e., physical, social, or psychological separation from individuals or groups) [43] in adolescents [44] and in older people [45], and the evidence shows that socially isolated people are more likely to suffer from suicidal thoughts [46] and depression [47].

Recognizably, there are studies showing associations between lifestyle factors and depression; thus, after the adjustment for age, sex, compliance with PA guidelines, BMI, smoking history, chronic disease status, financial perception, and sleep (quality and duration), there was an expected attenuation of the negative relation between SB domains and well-being outcomes. As a result, only the negative relation for occupation SB with GLS remained (*p* < 0.001), meaning that this relation was stronger and that it may be independent from the covariables included in the model.

### Findings in young adults

Our second goal was to identify whether the associations between SB domains and mental health outcomes differed with age. Our analyses indicated that leisure-time SB was negatively associated with PWB (*p* = 0.008) in young adults (18 to 24 years old), but we found no associations for the other SB domains.

One of the reasons why leisure-time SB may be negatively associated with PWB in young adults may be due to a reduction in PA during leisure time (competing with SB), which has been associated with higher levels of PWB with a positive impact on self-acceptance, positive relations with others and purpose in life [48]. In addition, the domain of PA is important. A study that evaluated the relation between different domains of PA and positive mental health in 456 men (mean age 29 years) indicated that only PA during leisure time was associated with better mental health, and no association was found for the commuting and occupational PA domains [49]. Another reason for leisure-time SB being negatively related to PWB may be that younger adults (recently emerged from adolescence) spend much of their leisure time in front of screens [22], as they are the largest users of social networks (such as Instagram® and TikTok®). In fact, a 2022 survey [50] reported that nearly all American teens (95%) have access to a smartphone (with few differences between income brackets [from 93% to < $30.000 to 96% > $75.000]), and 45% are "almost constantly" connected. Likewise, this phenomenon extends to young adults, where 96% of individuals from 18–29 years old report having a smartphone [51], with approximately 70% of them indicating daily use. Although not all studies corroborate the negative impact of social media on the PWB and mental health of young people [52, 53], with its use being important and facilitating social connection and support [54], it can certainly harm PWB, mainly depending on how, with whom, and why social media is used [55].

In addition, recent investigations have shown that younger people are the most exposed to various types of content, potentially criminals (cyberbullying, cyber-dating violence, sextortion, sexting, revenge porn, online dating, catfishing, and scammers) [56], with potential consequences on mental health [57]. Finally, in the younger group (18 to 24 years), the time spent in occupational SB was not associated with well-being outcomes, even acknowledging that they were the group spending more time in occupational SB (i.e., spending more 57 min/day than adults). This can possibly be justified by the younger adults of our sample, being predominantly students, spending its time in an occupational, educational environment, which can be perceived as a time dedicated to a purpose and direction in life, as a choice task that will bring meaning and benefits to society and themselves, while the adults are predominantly employees (staff and teachers).

### Findings in adults

In adults (≥ 25 years), after all the adjustments, occupational SB was negatively associated with GLS (*p* < 0.001) and MCS (*p* = 0.027), and transport-related SB was negatively associated with PWB (*p* = 0.017). In contrast to what happened in the younger group, leisure-time SB was not associated with well-being outcomes. Our cross-sectional observations are in line with the results from an experimental study with 146 office workers that assessed the efficacy of SB reduction through the use of standing desks versus a control group, while using objective measures and found a significant reduction in sitting time (-83.28 min/working day after 12 months) with repercussions in a reduction in daily anxiety after 6 and 12 months and an improvement in quality of life [58], providing insights that sitting time in the work setting can be harmful to mental health in adults. In addition, an investigation with more than 44,978 employees indicated that prolonged sitting time without breaks at work is associated with an increased risk of self-reported overall poor health and back/neck pain, important components of physical health, which can impact the quality of life by reducing well-being [59].

In our analysis, transport-related SB was associated with a reduction in PWB in adults, even though they spent less time commuting (≅ 21 min/day) than younger adults. Previous studies have explored the relationship between time spent commuting while sedentary and several measures of life satisfaction in workers (satisfaction with work, home, personal life and leisure time) and reported a negative effect of commuting on all variables above, especially for workers with lower income who live in areas of high population density [60] and thus are more exposed to traffic jams. In this case, in addition to being exposed to the negative effects of SB “per se”, the added stress of lack of control over time and the transfer of time with family and friends or other activities could reduce PWB, according to resource drain theory (i.e., the transfer of finite personal resources, such as time, attention, and energy, from one domain to another) [61].

It should be noted that leisure-time SB was not related to any of the measures of well-being and quality of life in our investigation, possibly because adults in our sample may be less likely to be involved in the situations mentioned in topic 4.1, which leads younger adults to have worse PWB. In addition, what may contribute to this nonrelation is the fact that our sample of adults spent significantly less time in leisure time compared to young adults (170 min/week versus 269 min/week, respectively).

## Strenghts and limitations

Although our study provides new insights into the relation between domains of SB and well-being/quality of life by age group, the main limitation was the cross-sectional nature of the data, which does not allow for the inference causality. In addition, by using questionnaires, we are more prone to recall and social desirability, which can result in an underestimation of SB. Another problem is that our sample failed to respond to many questions (missing data), which caused a reduction in the initial sample. However, we must mention as a strength the fact that this investigation is one the first to assess the relationship between several SB domains and well-being measures through a positive perspective (that is, not only analysing the associations with mental disorders but also with well-being) and considering distinct age groups, which, as shown, may possibly explain some of the prior inconsistent findings on this topic. Besides that, the exploration of the domains (or contexts) are in line with recent recommendations for public health [62].

### Theorical and practical and implications

Reducing physical inactivity in the global population has been a challenge, and the associate burden of chronic and mental diseases continues to increase [63, 64], thus, to understand how each specific SB can impact health will assist health entities to provide more accurate recommendations, an endeavour of key importance in an industrialized, technologic world discouraging the need for PA. Besides that, this investigation can supply for further insights about the importance of measuring SB and PA by domain through a combination of accelerometers (objective assessments) and questionnaires (subjective assessments). Considering aging, and although this was not the focus of this research more free time after retirement may represent and additional peril for spending more time in leisure SB, in front of screens. However, more research is necessary, since the association of technology use and well-being in the elderly is unclear [65] and, as cited before, the assessment of the SB domain is still less investigated.

## Conclusion

Our data indicate that although SB has already been recognized as a harmful behavior for well-being and quality of life, future studies should focus on understanding the domain in which SB is accumulated. Thus, although the current recommendations for SB are "sit less" or “minimize time sitting in front of screens”, our findings suggest that these recommendations should be more domain-oriented depending on age group. For younger adults, the domain of SB that seems to be more harmful to well-being is the leisure context, whereas in adults, the occupational context is more harmful. However, this must be confirmed in future experimental studies.

## Data Availability

Data that has been anonymized is available for research purposes upon request to Marlene Nunes Silva p4248@ulusofona.pt.
